# A case study of SMART attributes: a qualitative assessment of generalizability, retention rate, and trial quality

**DOI:** 10.1186/s13063-016-1368-3

**Published:** 2016-05-14

**Authors:** Erica E. M. Moodie, James C. Karran, Susan M. Shortreed

**Affiliations:** Department of Epidemiology, Biostatistics, and Public Health, McGill University, Purvis Hall, 1020 Pine Ave West, Montreal, QC H4B 2V2 Canada; Biostatistics Unit, Group Health Research Institute, 1730 Minor Avenue, Suite 1600, Seattle, WA 98101 USA

**Keywords:** Generalizability, Randomized trial, Retention, Schizophrenia, Sequential randomization, Antipsychotic medication

## Abstract

**Background:**

Personalizing medical care is becoming increasingly popular, particularly mental health care. There is growing interest in formalizing medical decision making based on evolving patient symptoms in an evidence-based manner. To determine optimal sequencing of treatments, the sequences themselves must be studied; this may be accomplished by using a sequential multiple assignment randomized trial (SMART). It has been hypothesized that SMART studies may improve participant retention and generalizability.

**Methods:**

We examine the hypotheses that SMART studies are more generalizable and have better retention than traditional randomized clinical trials via a case study of a SMART study of antipsychotic medications. We considered the Clinical Antipsychotic Trials of Intervention Effectiveness (CATIE) schizophrenia study, comparing the trial participant characteristics and overall retention to those of comparable trials found via a review of all related trials conducted from 2000 onwards.

**Results:**

A MEDLINE search returned 6435 results for primary screening; ultimately, 48 distinct trials were retained for analysis. The study population in CATIE was similar to, although perhaps less symptomatic than, the study populations of traditional randomized clinical trials (RCTs), suggesting no large gains in generalizability despite the pragmatic nature of the trial. However, CATIE did see good month-by-month retention.

**Conclusions:**

SMARTs offer the possibility of studying treatment sequences in a way that a series of traditional RCTs cannot. SMARTs may offer improved retention; however, this case study did not find evidence to suggest greater generalizability using this trial design.

**Trial registration:**

ClinicalTrials.gov NCT00014001. Registered on 6 April 2001.

**Electronic supplementary material:**

The online version of this article (doi:10.1186/s13063-016-1368-3) contains supplementary material, which is available to authorized users.

## Background

Individualizing medical decision making by implementing evidence-based treatment strategies is gaining in popularity. As noted by Murphy [11] and Robins [14], personalizing treatment choices based on evolving patient characteristics can be operationalized by constructing dynamic treatment regimes or adaptive treatment strategies using data collected from clinical trials or observational studies. A new paradigm was recently proposed to study dynamic treatment regimes: *Sequential multiple assignment randomized trials* (SMARTs) (as seen in the work of [1, 3, 6, 7, 13]). In a SMART design, participants are initially randomized to one of a set of treatment options; individuals may then be re-randomized at subsequent treatment stages (i.e., at later decision points).

The sequential randomization approach of the SMART paradigm is required to determine best treatment strategies when considering long-term outcomes, because treatment interactions and delayed effects cannot be detected in traditional randomized clinical trials (RCTs) that study only one decision time in a treatment course. This property is well recognized as an advantage, and several methods (such as those detailed by [11, 12, 14, 18]) have been developed to construct adaptive treatment strategies using data collected from a SMART.

It is common for treatment options and randomization probabilities at each treatment stage in a SMART to reflect response to prior treatment. It has been hypothesized that sequential randomization and randomization probabilities that take into account patients’ prior responses to treatment, which are integral to SMART studies, may improve participant retention and generalizability of study results. Retention may be improved because participants may continue in the trial even if a particular treatment is not working for them: treatment changes are explicitly built into the protocol of a SMART design. SMART study results may be more generalizable, since there is value — from the scientific investigator’s point of view — to maximizing the heterogeneity of the participants. Participants who are “very alike” may not differ sufficiently in their responses to treatment to be able to detect any need for treatment tailoring. This often leads to broad enrollment criteria which could provide results applicable to a more general population.

While both hypotheses and their motivations are plausible, they have yet to be examined empirically. These as yet undetermined benefits were studied through a case study [16] that compared single stage trials investigating antipsychotic medication to a well-known SMART study, the Clinical Antipsychotic Trials of Intervention Effectiveness (CATIE) schizophrenia study. A systematic review of all randomized trials that investigated antipsychotic medication for the treatment of schizophrenia conducted in the same time period or subsequent to CATIE was undertaken.

## Sequential multiple assignment randomized trials

To illustrate SMARTs more concretely, a simplified version of CATIE, which has been described in detail elsewhere (by [15–17]), is provided. CATIE was a multisite study funded by the National Institute of Mental Health designed to evaluate the effectiveness of antipsychotic treatment strategies for patients with schizophrenia. Since CATIE was a practical clinical trial and the goal of the study was to assess real-world effectiveness, there were few exclusion criteria and patients could choose to transition into a new treatment stage (i.e., receive a different treatment) at any clinical visit in consultation with their clinician.

**Fig. 1:**
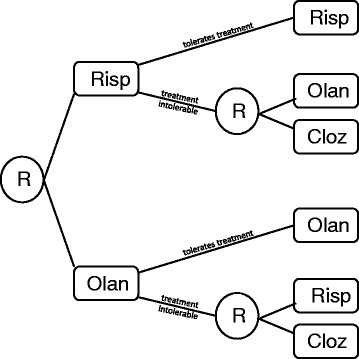
A simplified schematic of the CATIE study design. Circled *R*s represent randomization

Following recruitment, CATIE patients were randomized to receive one of five possible medications (perphenazine, olanzapine, quetiapine, risperidone, or ziprasidone). Patients were followed monthly and symptoms and quality of life measures were recorded. If the initial assigned medication was both tolerable (had acceptable side effects) and efficacious (provided adequate management of antipsychotic symptoms), the patient continued on this initial medication. If the patient wished to discontinue the medication, he was randomized to one of five possible second-stage treatments (clozapine, olanzapine, quetiapine, risperidone, or ziprasidone). CATIE stage-two randomization probabilities took into account prior treatment and reason for discontinuation. For example, individuals who discontinued a medication in the first stage were not re-randomized to that medication in the second stage. Similarly, if an individual discontinued a medication due to side effects, he/she could not be randomized to clozapine, which is a highly efficacious antipsychotic with a significant side effect profile (Fig. 1). CATIE participants were followed for an 18-month period with final health outcomes measured at the conclusion of the trial.

## Methods

### Search strategy and selection criteria

This review was conducted in accordance with the Preferred Reporting Items for Systematic Reviews and Meta-Analyses (PRISMA) statement on the transparent reporting of systematic reviews and meta-analyses. Ovid was used to search MEDLINE (including In-Process and Other Non-Indexed citations) for RCTs performed in the same time frame as and subsequent to CATIE. The search string was developed with the assistance and input of a professional librarian (see Additional file 1 for details).

Specifically, the search was for RCTs conducted from January 1, 2000 onwards, conducted on patients with schizophrenia, and involving neuroleptic drugs studied in CATIE (aripiprazole, clozapine, fluphenazine, olanzapine, perphenazine, quetiapine, risperidone, or ziprasidone). Although the search specified trials from January 2000 onwards, ultimately only trials initiated on January 1, 2001 or later were included since the CATIE study began in December 2000. Trials were excluded if they involved non-oral drug formulations (such as long-acting injectable antipsychotic preparations), or if they studied healthy participants (i.e., individuals without schizophrenia) or participants experiencing a first episode of schizophrenia, again aiming to mimic the CATIE study goals. Only trials reported in English were included. If a trial’s suitability for inclusion could not be determined from the title and abstract, the full article was retrieved to obtain the relevant information.

### Data abstraction and analysis

Abstracted information from included RCTs was guided by the CATIE study design and the information collected from CATIE participants at baseline. Basic trial design parameters were recorded: drug treatments in each trial arm, total number of participants, number of participants by trial arm, primary outcome, planned follow-up duration (in weeks), blinding (double-blinded, single-blinded, or open-label), and treatment location (in hospital, community-based, etc.). Information on the trials’ inclusion/exclusion criteria was also recorded, including age for inclusion, recruitment country, recruitment locations (from home, from inpatient hospitalization, etc.), and general inclusion criteria (a wide-ranging category that predominantly recorded which diagnostic manual had been used to determine the diagnosis of schizophrenia and whether trial participants were considered to be treatment-resistant). Exclusion criteria were separated into medical and psychiatric comorbidities, criteria based on concurrent treatments, and “other” criteria (a broad category including past drug/medical history, use of contraception, spoken language, educational level, etc.). In addition to design features, data were collected on total retention rates (number and percentage of participants still participating in the trial at the study endpoint).

Abstracted patient characteristics included racial demographics (number and percentage of participants who were white/Caucasian, black/Afro-Caribbean, or other), age of participants (minimum, maximum, and mean age), baseline body weight in kilograms, age at onset of schizophrenia, years since diagnosis of schizophrenia, percentage of treatment-naïve participants at baseline, sex, educational attainment, and employment status. Mean age and time since diagnosis were combined to determine mean age at onset for comparative purposes.

When available, the mean and standard deviation of psychiatric and psychological test scores at baseline were abstracted; these are described below. The Positive and Negative Syndrome Scale (PANSS) is a common measure of psychotic symptoms in patients with schizophrenia, which ranges from 0 to 270 with a higher score indicating more severe symptoms. Clinical Global Impression (CGI) Scale scores range from 1 to 7 with a higher score indicating more severe illness. The Montgomery-Asberg Depression Rating Scale (MADRS) ranges from 0 to 60 with higher scores indicating more severe depressive symptoms. While this depression measure was commonly reported in other trials, CATIE used the Calgary Depression Scale, which ranges from 0 to 27 with higher scores indicating more severe depressive symptoms. For comparison purposes, note that an individual who scores 6 or less on the MADRS would be classified as having absent symptoms, 7–9 mild symptoms, 20–34 moderate symptoms, and greater than 34 severe symptoms. As described by Müller et al. [10], individuals with scores of 3 or less on the Calgary Depression Scale are classified as absent of symptoms, 3–6 as mildly depressed, 6–10 as moderately depressed, and greater than 10 as severely depressed. When available, means and standard deviations of several scales for measuring extrapyramidal symptoms (EPS), which include ticks and tremors and are side effects of some antipsychotic medications, were abstracted. The Simpson-Angus Scale (SAS, range 0–40), the Barnes [2] Akathisia Rating Scale (BARS, range 0–9), and the Abnormal Involuntary Movement Scale (AIMS, range 0–14) all use higher scores to indicate more severe EPS.

Finally, following the suggestion of a referee, we used the papers as well as information available through trial registration at the website ClinicalTrials.gov to assess the trial quality as measured by the risk of bias using the Cochrane Collaboration tool [5].

It was not possible to perform inferential analyses to assess generalizability to the high level of missing information (i.e., published trial materials not reporting information); thus, the analysis in this case study was qualitative in nature. Sample size, retention, and baseline characteristics of CATIE participants to participants of the trials included in this review were descriptively compared, with a specific focus on PANSS scores (mean and variability) since this is the primary measure of antipsychotic symptoms in this population. Symptom severity can affect retention in many ways and can greatly impact the generalizability of a trial’s results.

To examine retention rate, we used a Kaplan-Meier approach, plotting the month-by-month retention rate in CATIE alongside the end-of-study retention rate for each traditional clinical trial. By-month retention rate for the traditional RCTs was unavailable; however, the median RCT length was 8 weeks, and so the by-month Kaplan-Meier curves for attrition for most traditional RCTs would be quite uninformative. The by-month analysis of retention has not, to our knowledge, been used previously to compare retention rate across trials; however, a similar approach of studying retention rates within trials by participant characteristics using Kaplan-Meier curves has been employed [4, 8].

## Results

Our initial MEDLINE search returned 6435 papers reporting the results of trials using our primary screening of titles and abstracts, of which 198 (3.1 %) were eligible for a secondary screening of the publication’s full text. The main reason for unsuitability for secondary screening was that the publication did not report on an RCT (see Additional file 1 for further details). Of the papers that received a secondary screening, 57 (28.8 %) publications, corresponding to 48 distinct trials, met the required criteria.

Some differences were found between the inclusion and exclusion criteria for CATIE and the abstracted traditional RCT. Similar to CATIE, traditional RCTs conducted in adults included a wide age range (typically 18–65 or 18 and older). On the other hand, only 9 of the 20 trials reporting recruitment location did so from both inpatient and outpatient settings; CATIE recruited from a wide range of settings including teaching hospitals, Veterans Affairs clinics, academic institutions, and private hospitals. The most common exclusion criteria in the traditional RCTs were substance abuse, suicide risk, and epilepsy. In CATIE, the primary exclusion criterion was severe treatment-refractory (treatment-resistant) schizophrenia.

On comparison with the abstracted traditional RCTs, CATIE was one of the largest clinical trials of patient with schizophrenia, with only one of the traditional RCTs enrolling more patients. CATIE participants were of a similar age (mean = 40.5 years) compared to those in traditional RCTs; the interquartile range (IQR) of the mean age across the traditional RCTs conducted among adults was 35.7–40.8 years. The age at onset among CATIE participants (26.0 years) was comparable to that of traditional RCTs conducted among adults, where the IQR of the mean age at onset was 25.5–29.0 years. CATIE enrolled more male participants than the traditional RCTs (75 % male in CATIE, compared to an IQR across traditional RCTs of 53–69 %). Sixty percent of CATIE participants were non-white compared to 50.0–83.5 % of participants in traditional RCTs. CATIE participants tended to have slightly lower PANSS scores (mean PANSS score at enrollment = 75.7 versus IQR of 83.4–97.9) and CGI scores (mean CGI score at CATIE enrollment = 4.0 versus IQR of 4.6–5.1). Additionally, CATIE participants were more likely to be treatment-naïve than participants in the traditional RCTs evaluating treatment of schizophrenia (43.0 % versus 18.7–40.0 %). Further comparison results are provided in tabular form in Additional file 1.

Several characteristics were reported too infrequently to permit any meaningful comparison between CATIE and the traditional RCTs. For example, education level and employment status were reported in only three RCTs. The MADRS, SAS, and AIMS were each reported by two trials, and BARS was reported by only one trial.

**Fig. 2:**
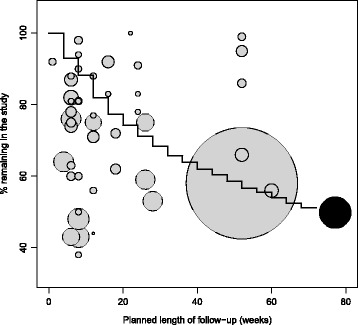
Retention rate in CATIE and traditional RCTs of patients with schizophrenia by the planned length of follow-up of the trial. Circle radius is a function of the total number enrolled in the trial (for reference the CATIE trial enrolled 1460 participants). The black line indicates the follow-up rate by month in CATIE, with retention rate at the 18-month CATIE follow-up indicated by the black circle

**Fig. 3:**
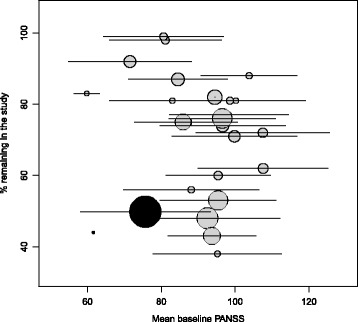
Retention rate in CATIE and traditional RCTs of patients with schizophrenia versus mean PANSS score at study enrollment. Location of circle on horizontal axis indicates mean PANSS score at baseline, and circle radius is a function of the total number enrolled in the trial; circle color indicates the planned length of follow-up. The horizontal bars indicate +/- one standard deviation of PANSS scores at study enrollment. The CATIE study is represented by the black circle

CATIE has a favorable retention profile compared to traditional RCTs (Fig. 2), when compared month by month. While 50.0 % of CATIE participants withdrew or were lost to follow-up by the end of planned follow-up of 18 months, the by-month attrition rate was such that retention was higher than that in most other RCTs at any given month. There was no relationship between initial mean PANSS score of a study’s participants and the study’s retention rate (Fig. 3).

Trial quality was ascertained for all trials in the study. Twelve traditional RCTs were deemed to be at high risk of bias due to lack of blinding in study treatments, while a further 32 were deemed to be of “uncertain” risk, primarily due to the inability to ascertain the method of sequence generation for randomization and concealment of allocation. Of the 12 trials that did not feature blinding, only two did so because clozapine, a drug whose usage which requires careful monitoring and regular blood tests due to a risk of agranulocytosis, was included as a treatment arm. CATIE also included clozapine as a possible treatment arm; treatment with clozapine in CATIE was open-label, whereas all other treatment allocations were concealed and double-blinded. With only two traditional RCTs being graded as at low risk of bias, a subgroup analysis based on this measure was deemed infeasible. Qualitatively, however, it appears that CATIE is among a small number of trials that were reported in sufficient detail to ascertain that the risk of bias was low.

## Discussion

A case study was performed to examine whether the CATIE SMART study enrolled a population that was more generalizable or enjoyed better retention rates than traditional RCTs designed to evaluate similar antipsychotic medications for patients with schizophrenia. CATIE is one of the first and largest SMARTs to be conducted. Due to differences in variables reported, generalizability was difficult to assess quantitatively, and so a qualitative comparison was performed. The CATIE protocol listed very few exclusion criteria compared to the traditional RCTs; however, there is insufficient evidence to conclude SMART studies are inherently more generalizable or attract a more diverse participant pool.

An alternative approach to assessing generalizability would have been to compare the distribution of CATIE’s participant characteristics with the distribution of characteristics of the population of people living with schizophrenia. However, this, too, proved not to be possible given the information available in the literature. The incidence of schizophrenia is higher in men than women and onset peaks in the range of 15–24 years, although in women there is a secondary peak in the years 55–64 [9]. Incidence rates appear to vary by ethnicity [9]; however, information on the global or even United States-specific ethnic composition of people living with schizophrenia is not reported.

CATIE exhibited a higher than typical retention rate when viewed on a monthly basis. One might speculate that this is a feature that would be shared by other SMARTs, given the inherent design which allows participants to change away from ineffective or intolerable treatments without disenrolling from the study. Further research should consider whether these findings hold across a range of other domains in which SMARTs have been conducted.

## Conclusions

SMARTs offer the possibility of studying treatment interactions and delayed effects, and they may provide a better assessment of “real-world” performance of treatment sequences than traditional RCTs. The CATIE study exhibited very low risk of bias. CATIE also possessed a by-month retention rate that was higher than that typically observed in the traditional RCTs conducted in the same time period to assess the same treatments for the same condition. The SMART design may offer improved retention compared to traditional trial designs; however, this case study did not find evidence to suggest greater generalizability using the SMART approach.

## Additional file

Additional file 1: PDF document entitled “Supplementary Materials” detailing Medline search strings and the complete study selection protocol, and containing two tables summarizing the included RCTs (planned length of follow-up, enrolled sample size, and retention rate) and participant characteristics at study enrollment. (DOCX 40 kb)

## Abbreviations

AIMS: Abnormal Involuntary Movement Scale
BARS: Barnes Akathisia Rating Scale
CATIE: Clinical Antipsychotic Trials of Intervention Effectiveness
CGI: Clinical Global Impression
EPS: extrapyramidal symptoms
MADRS: Montgomery-Asberg Depression Rating Scale
PANSS: Positive and Negative Syndrome Scale
RCT: randomized clinical trial
SAS: Simpson-Angus Scale
SMART: Sequential multiple assignment randomized trial
