# Supplementary material for: A case study of SMART attributes: a qualitative assessment of generalizability, retention rate, and trial quality
Source: Trials. 2016 May 14;17:242. doi: 10.1186/s13063-016-1368-3 (PMC4894372; doi:10.1186/s13063-016-1368-3)
Supplement: Supplementary file 1 — PDF document entitled “Supplementary Materials” detailing Medline search strings and the complete study selection protocol, and containing two tables summarizing the included RCTs (planned length of follow-up, enrolled sample size, and retention rate) and participant characteristics at study enrollment. (DOCX 40 kb) [file 13063_2016_1368_MOESM1_ESM.docx]

**Supplementary Materials Search Strings and Study Selection Strategy**

**Medline search strings**

1. exp Schizophrenia/
2. schizo*.mp.
3. 1 or 2
4. clozapin*.mp.
5. clozaril*.mp.
6. leponex*.mp.
7. 4 or 5 or 6
8. olanzapin*.mp.
9. zyprex*.mp.
10. zypadhera*.mp.
11. symbyax*.mp.
12. 8 or 9 or 10 or 11
13. perphenazin*.mp.
14. trilafon*.mp.
15. decentan*.mp.
16. etrafon*.mp.
17. triavil*.mp.
18. 13 or 14 or 15 or 16 or 17
19. quetiapin*.mp.
20. seroquel*.mp.
21. 19 or 20
22. risperidon*.mp.
23. risperdal*.mp.
24. 22 or 23
25. ziprasidon*.mp.
26. geodon*.mp.
27. zeldox*.mp.
28. 25 or 26 or 27
29. aripiprazol*.mp.
30. abilitat*.mp.
31. abilify*.mp.
32. 29 or 30 or 31
33. fluphenazin*.mp.
34. prolixin*.mp.
35. 33 or 34
36. 7 or 12 or 18 or 21 or 24 or 28 or 32 or 35
37. randomized controlled trial.pt.
38. controlled clinical trial.pt.
39. randomized.ab.
40. placebo.ab.
41. drug therapy.fs.
42. randomly.ab.
43. trial.ab.
44. groups.ab.
45. or/37-44
46. 3 and 36 and 45
47. limit 46 to yr=“2000 -Current”
48. 46 and 47
49. 48 not (exp animals/ not humans.sh.)

**Study selection**

Our initial MEDLINE search returned 6435 results for primary screening, of titles and abstracts. One hundred and ninety eight of these were found eligible for a secondary screening, of the full text of the publications. Of these 198, 57 publications (corresponding to 48 distinct trials) passed the secondary screening. In total 113 trials did not meet the inclusion criteria. The reasons for failing to meet the criteria of the second screening were as follows; trials initiated outside the range of our inclusion period (N=57), participants with non-schizophrenia diagnoses (N=41), insufficient details of a relevant trial (N=4), participants with first-episode schizophrenia (N=4), data derived from the CATIE Project (N=2), papers of a meta-analytic nature/describing multiple pooled trials (N=2), trial not classifiable as a randomized controlled trial (N=1), study involving healthy participants (N=1), excluded drug or treatment (N=1).

Thirteen trials were unsuitable for inclusion, as we were unable to ascertain the dates during which the trials were carried out. Authors were contacted up to 3 times and, if no reply was received, the trial was not included. Fifteen trials were unsuitable for inclusion as the paper we were unable to obtain the original paper to determine whether the necessary criteria for inclusion were met. Results from our MEDLINE search were exported to RefWorks. Duplicates were removed, having been identified with RefWorks tools for highlighting duplicate entries. Titles and abstracts were screened according to the criteria given below.

*First Screening*

At the first screening, our inclusion criteria were as follows:

1. Randomized controlled trials

2. Trials performed between January 1, 2001 and March 14, 2014. Where this is unclear, but trial otherwise fits inclusion criteria, the publication will be retained so that the full document can be read and the trial dates determined.

3. Trials involving participants with a confirmed diagnosis of schizophrenia (any subtype), both chronic, acute, or combination of both.

4. Trials involving treatment with any of the following anti-psychotic drugs (oral only), alone or in combination: Aripirazole, Clozapine, Fluphenazine, Olanzapine, Perphenazine, Quetiapine, Risperidone.

5. Trials with any primary outcome measure (BPRS, PANSS, Custom psychopathology rating scales, etc.) will all be retained, provided that the trial relates to the use of a relevant drug in participants with schizophrenia.

6. Publications in English only.

The exclusion criteria were:

1. Studies will be excluded if they describe anything other than a randomized controlled trial, e.g. case-cohort, case-control, case-series, case report, cohort, cross-sectional, simulations, etc. Search results will also be excluded if they do not describe a study but instead relate to some other element of a journal such as News, review article, correspondence, etc.

2. Trials involving any other psychiatric disease other than schizophrenia. This excluded schizophrenia-related disorders such as schizoaffective disorder, schizotypal personality disorder, and generalized psychosis from any cause.

3. Trials comparing participants with schizophrenia to healthy control subjects.

4. Trials studying schizophrenia in first-episode patients.

5. Trials involving long-acting injectable forms of anti-psychotics, such as intramuscular depot preparations.

Of the 6435 papers found by our rather broad search criteria, 96.9% of search results were discarded at the primary screening as they did not in fact meet the criteria for comparability with CATIE. A large number (N=1784; 27.7%) were not RCTs and included cross-sectional and retrospective studies. Another large group of papers (N=1222; 18.9%) were excluded because they were not formal papers on a specific RCT but were other published material, such as letters or editorials. Several papers (N=1102; 17.1%) were excluded based on our primary screen; example reasons of exclusion include: the paper described studies that investigated a condition other than schizophrenia, treatments under study were not under consideration for this review, the patient population included healthy participants. The remaining papers that were excluded (N=2129; 33.1%) were not published in English, did not have at least two treatment groups, or were review articles.

*Second Screening:*

A second screening of the articles was performed on the following basis:

1. The full texts of all trials whose titles and abstracts either indicate that they are suitable for inclusion, or that meet the inclusion criteria while obviously meeting any of the exclusion criteria, will be retrieved for a secondary screening.

2. In the secondary screening the full article will be read to ensure that the trial is valid for inclusion. Where information (such as trial dates) is missing the corresponding author of the paper will be contacted, to allow these missing details to be determined.

3. In the case that no reply is forthcoming, or that the correspondence details are inaccurate or out of date, the relevant paper will be excluded.

The 113 manuscripts that were excluded in the secondary screening were omitted for the following reasons: the trials were initiated outside the range of our inclusion period (N=57; 28.8%), participants did not have schizophrenia diagnoses (N=42; 21.3%), manuscripts provided insufficient details of the trial (N=4; 2.0%), trial participants were included those with first-episode schizophrenia (N=4; 2.0%), the paper described data collected from the CATIE Project (N=2; 1.0%), the paper was meta-analytic in nature/described multiple pooled trials (N=2; 2.0%), the study was not an RCT (N=1, 0.5%) or the trial involved a drug/treatment that did not correspond with our inclusion criteria. (N=1, 0.5%).

**Table S1.** Included studies: Planned length of follow-up, enrolled sample size, and retention rate.

| Study name & key publications | Years of study | Planned follow-up (weeks) | Total sample size | Retention:  No. (%) | Recruitment Country | Recruitment locations |
| --- | --- | --- | --- | --- | --- | --- |
| CATIE | 2000-2004 | 77 | 1460 | 929 (50%) | USA | In-, out-patient |
| Yamashita H. et al. (2004)^1^, Yamshita H. et al. (2005)^2^ | 2001 | 8 | 92 | 90 (98%) | Japan | In-patient |
| Zhong K. et al.^3^ | 2001-2002 | 8 | 673 | 322 (48%) | USA |  |
| Lin C. et al (2013)^4^ | 2001-2002 | 12 | 59 | 52 (88%) |  |  |
| Wagner M. et al.^5^ | 2001-2003 | 8 | 52 | 26 (50%) | Germany | In-patient |
| Chen J. et al.^6^ | 2001-2004 | 8 | 48 | 39 (81%) | Taiwan |  |
| Riedel M. et al (2007-a)^7^ | 2001-2004 | 8 | 52 | 20 (38%) |  | In-patient |
| Riedel M. et al (2007-b )^8^ | 2001-2004 | 12 | 44 | 34 (77%) |  | In-, out-patient |
| Jakovlevic M. et al.^9^ | 2002-2003 | 22 | 22 | 22 (100%) | Croatia |  |
| ZODIAC^10^,^11^ | 2002-2006 | 52 | 18,154 | (58%) | Multi-country (Multi-continent) | In-, out-patient |
| Freudenreich O. et al.^12^ | 2002-2005 | 6 | 24 |  |  |  |
| Kryzhanovskaya L. et al.^13^,  Kemp D. et al.^14^ | 2003 | 6 | 107 | 64 (60%) | USA and Russia | In-, out-patient |
| Berwaerts J. et al.^15^ | 2003 | 1 | 76 | 70 (92%) |  |  |
| Apiquian R. et al.^16^ | 2003-2004 | 6 | 48 | 39 (81%) |  | In-, out-patient |
| Harvey P. et al. (2006) ^17^ | 2003-2005 | 8 | 673 | 289 (43%) |  | In-patient |
| MOZART^18^ | 2003-2005 | 18 | 147 | 90 (62%) | Italy |  |
| Suzuki H. et al.^19^ | 2003-2006 | 8 | 31 |  | Japan | In-patient |
| Suzuki T. et al.^20^ | 2003-2006 | 24 | 78 | 71 (91%) | Japan | In-, out-patient |
| Kane J. et al.^21^ | 2003-2007 | 28 | 566 | 301 (53%) | Multi-country (Multi-continent) | In-, out-patient |
| Marder S. et al.^22^ | 2004 | 6 | 444 | 194 (43%) |  |  |
| Findling R. et al. (2012)^23^ | 2004 | 6 | 222 | 164 (74%) |  |  |
| Gurpegui M. et al.^24^, Alvarez E. et al.^25^, Ciudad A. et al.^26^ | 2004 | 52 | 247 | 163 (66%) | Spain | Out-patient |
| Zhang H. et al.^27^ | 2004-2005 | 6 | 239 | 208 (87%) |  |  |
| Haas M. et al.^28^ | 2004-2005 | 6 | 160 | 125 (78%) | Multi-country (Multi-continent) |  |
| Khan R. et al.^29^ | 2004-2005 | 6 | 588 | 466 (76%) | Multi-country (Multi-continent) |  |
| STAR^30^, ^31^, ^32^ | 2004-2005 | 26 | 555 | 330 (59%) | Multi-country (Europe) | In-, out-patient |
| Harvey P. et al.^33^ | 2004-2006 | 18 | 130 | 94 (72%) | Italy |  |
| Okugawa G. et al.^34^ | 2004-2007 | 12 | 66 | 37 (56%) | Japan | In-, out-patient |
| Findling R. et al. (2008)^35^ | 2005 | 6 | 302 | 258 (82%) | Multi-country (Multi-continent) |  |
| Voruganti L. et al.^36^ | 2005 | 52 | 86 | 85 (99%) | Canada |  |
| Cutler A. et al.^37^ | 2005-2006 | 4 | 593 | 381 (64%) |  |  |
| QUERISOLA^38^ | 2005-2007 | 8 | 75 | 61 (81%) | Italy |  |
| Maat A. et al.^39^ | 2005-2011 | 8 | 80 | 48 (60%) | Netherlands |  |
| Chang J. et al.^40^ | 2006 | 8 | 62 | 56 (90%) | Korea |  |
| Fleischhacker W. et al.^41^ | 2006 | 16 | 207 | 190 (92%) |  |  |
| SCoP^42^, ^43^, ^44^ | 2006 | 52 | 106 | 91 (86%) | Italy |  |
| De Hert M. et al.^45^ | 2006-2007 | 60 | 261 | 147 (56%) | Multi-country (Europe) |  |
| Tybura P. et al.^46^ | 2006-2010 | 12 | 191 | 136 (71%) | Poland |  |
| Schreiner A. et al.^47^ | 2007-2009 | 26 | 459 | 346 (75%) | Multi-country (Multi-continent) |  |
| Ghalieha A. et al.^48^ | 2008 | 8 | 51 | 48, 94% | Iran | Psych. in-patient |
| Muscatello M. et al. (2011)^49^ | 2008-2009 | 24 | 40 | 31 (78%) | Italy | Out-patient |
| Lin C. et al. (2010)^50^ | 2009 | 6 | 88 | 55 (63%) | China |  |
| Li Y. et al.^51^ | 2009 | 6 | 119 | 89 (75%) |  | In-patient |
| Jindal K. et al.^52^ | 2009 | 6 | 60 | 53 (88%) | India | In-patient |
| Kwon J. et al.^53^ | 2009 | 12 | 389 | 293 (75%) | Multi-country (Asia) |  |
| SEROLA^54^ | 2009 | 12 | 9 | 4 (44%) | Denmark and Sweden |  |
| Lee B. et al.^55^ | 2009-2010 | 24 | 35 | 29 (83%) | Korea | In-patient |
| GiSAS^56^ | 2010 | 52 | 180 | 170 (95%) | Italy |  |
| Muscatello M. et al. (2014)^57^ | 2011-2012 | 16 | 40 | 33 (83%) |  |  |

**Table S2.** Participant characteristics at study enrollment: BPRS: Brief Psychiatric Rating Scale ranges from 0 to 126 with a higher score indicating more severe symptoms, PANSS: Positive and Negative Syndrome Scale ranges from 0 to 270 with a higher score indicating more severe symptoms, CGI: Clinical Global Impression scale ranges from 1 to 7 with higher scores indicating more severe illness.

| Study name & key publications | Age at baseline:  Mean in years (SD) | Sex: Male | Race:  White/  Cauc. | Race:  Black/  Afro-  Carib. | Race:  Other | Weight, kg: Mean (SD) | Age at onset: Mean (SD) | Time since dx:  Mean (SD) | Tmt naïve | BPRS: Mean (SD) | PANSS: Mean (SD) | CGI: Mean (SD) |
| --- | --- | --- | --- | --- | --- | --- | --- | --- | --- | --- | --- | --- |
| CATIE | 40.5 (11.1) | 1080  (74%) | 874  60% | 513  35% | 73  5% | 88.7 (21.4) | 26.0  (8.8) |  | 43  (3%) |  | 75.7 (17.6) | 4.0 (0.9) |
| Yamashita H. et al. (2004)^1^, Yamshita H. et al. (2005)^2^ | 59.9 (10.5) | 48 (52%) |  |  |  |  |  | 34.0 (9.5) |  |  | 81.1 (15.2) |  |
| Zhong K. et al.^3^ | 39.9 (10.8) | (76%) | 38% | 51% | 10% | 86.6 (21.4) |  |  |  |  | 92.5 (19.6) | 4.6 (0.7) |
| Lin C. et al (2013)^4^ |  |  |  |  |  |  |  |  |  |  |  |  |
| Wagner M. et al.^5^ |  |  |  |  |  |  |  |  |  |  |  |  |
| Chen J. et al.^6^ | 37.0 (11.4) | 28 (58%) |  |  |  |  | 25.9 (8.9) | 11.2 (9.2) |  |  | 83 (17) |  |
| Riedel M. et al (2007-a)^7^ | 35.6 (11.7) |  |  |  |  | 77.7 (12.9) |  | 6.6 (8.2) | 21 (40%) |  | 95.2 (17.4) | 5.5 (0.7) |
| Riedel M. et al (2007-b )^8^ | 34.4 (11.6) |  |  |  |  |  |  | 2.8 (9.5) |  |  | 98.3 (16.1) |  |
| Jakovlevic M. et al.^9^ |  |  |  |  |  |  |  |  |  |  |  |  |
| ZODIAC^10^, ^11^ | 41.1 (13.1) | 9,761 (55%) | 10,797 (60%) | 3,097 (17%) | 4,151 (23%) |  |  | 10.4 (10.8) |  |  |  | 4.2 (1.0) |
| Freudenreich O. et al.^12^ | 42.3 | (88%) |  |  |  |  |  |  |  |  | 75 |  |
| Kryzhanovskaya L. et al.^13^, Kemp D. et al.^14^ | 16.1 (1.3) | 75 (70%) | 77 (72%) |  |  |  | 13 (3) |  |  | 50.2 (9.3) | 95.4 (14.1) | 4.9 (0.8) |
| Berwaerts J. et al.^15^ | 36.7 (9.9) |  | 74 (97%) |  | 2 (3%) |  |  |  |  |  |  |  |
| Apiquian R. et al.^16^ | 30.8 (9.0) | 22 (51%) |  |  |  |  |  | 6.3 (7.1) | 8 (18.7%) |  | 100.2 (18.8) |  |
| Harvey P. et al. (2006)^17^ | 40.0 (10.7) | (73%) | 50.00% | 41.00% | 9.00% |  |  |  |  |  |  |  |
| MOZART 22821 ^18^ | 40 (11) | 101 (69%) |  |  |  | 82.4 (17.6) |  | 13.8 |  |  | 107.6 (17.6) | 5.2 (0.7) |
| Suzuki H. et al.^19^ | 56.7 (9.7) | 15 (48%) |  |  |  |  |  | 29.8 (10.3) |  | 36.2 (5.2) |  |  |
| Suzuki T. et al.^20^ | 44.9 (15.2) | 35 (45%) |  |  |  |  |  | 17.0 (11.7) | 15 (19%) | 72.6 (8.5) |  |  |
| Kane J. et al.^21^ | 37.8 (10.5) | 384 (68%) | 168 (30%) | 177 (31%) | 219 (39%) |  | 22.8 (7.7) |  |  |  | 95.4 (15.7) | 4.8 (0.7) |
| Marder S. et al.^22^ | 41.6 | (74%) |  |  |  |  |  |  |  |  | 93.7 (11.9) |  |
| Findling R. et al. (2012)^23^ | 15.4 (1.3) | 129 (59%) | 135 (61%) | 27 (12%) | 58 (27%) | 62.5 (14.4) | 13.6 (3.2) | 2.4 (2.1) |  |  | 96.6 (17.0) |  |
| Gurpegui M. et al.^24^, Alvarez E. et al.^25^, Ciudad A. et al.^26^ | 36.3 (10.6) | 179 (75%) |  |  |  | 77.2 (15.0) | 23.9 (7.0) |  |  |  |  |  |
| Zhang H. et al.^27^ | 34.8 (10.9) | 119 (50%) |  |  |  | 62.9 (11.1) |  |  |  | 46.2 (7.7) | 84.5 (13.4) |  |
| Haas M. et al.^28^ | 15.6 (1.3) | 102 (64%) | 84 (53%) | 15 (9%) | 61 (38%) |  | 14.7 (2.2) |  |  |  |  |  |
| Khan R. et al.^29^ | 34.2 (10.4) | (60%) | (59%) | (5%) | (36%) |  | 26.4 (8.4) | 8.3 (8.1) |  |  | 96.5 (14.4) | 4.9 (0.7) |
| STAR^30, 31^, ^32^ | 38.5 | 332 (60%) | 536 (96%) | 4 (1%) | 15 (3%) | 80.7 | 28.5 |  |  |  |  |  |
| Harvey P. et al.^33^ | 39.9 (10.5) | (69%) | 130 (100%) |  |  |  |  | 13.5 |  |  | 107.5 (18.1) |  |
| Okugawa G. et al.^34^ | 40.3 (15.9) |  |  |  |  | 59.3 (14.5) | 33.7 (14.8) |  |  |  | 88.1 (18.4) |  |
| Findling R. et al. (2008)^35^ | 15.5 (1.4) | 171 (57%) | 180 (60%) | 34 (11%) | 88 (29%) | 63.8 (16.7) | 14.1 (2.4) | 1.4 (2.0) | 26 (9%) |  | 94.5 | 4.6 |
| Voruganti L. et al.^36^ | 40 (14) | 63 (84%) |  |  |  |  |  | 14.7 (12) |  |  | 80.6 (16.2) |  |
| Cutler A. et al.^37^ | 39.9 (10.3) | 472 (70%) | 208 (35%) | 299 (50%) | 86 (15%) | 81.5 (17.6) |  |  |  |  |  |  |
| QUERISOLA^38^ | 39 (11) | 42 (56%) |  |  |  |  |  |  |  |  | 98.6 (20.2) |  |
| Maat A. et al.^39^ | 26.3 (6.7) | 64 (80%) | 54 (68%) | 26 (32%) |  |  |  |  | 46 (57.3%) |  |  |  |
| Chang J. et al.^40^ | 32.5 (7.9) | 48 (77%) |  |  |  |  | 19.8 (4.8) |  |  | 48.1 (10.0) |  | 4.1 (0.7) |
| Fleischhacker W. et al.^41^ | 39 (10.4) | 134 (65%) | 197 (95%) |  | 10 (5%) | 92.3 (17.3) |  | 13.5 (9.2) |  |  | 71.5 (16.6) |  |
| SCoP ^42^, ^43^, ^44^ | 40.9 (9.9) | 69 (65%) |  |  |  |  |  |  |  | 60 *(median)* |  |  |
| De Hert M. et al.^45^ | 35.9 | (47%) |  |  |  |  |  |  |  |  |  |  |
| Tybura P. et al.^46^ | 36.1 (12.4) |  |  |  |  |  | 26.2 (7.4) |  |  |  | 99.8 (16.9) |  |
| Schreiner A. et al.^47^ | 38.2 (11.3) | (58%) |  |  |  |  |  | 10.7 (9.6) |  |  |  |  |
| Ghalieha A. et al.^48^ | 34.06 (8.4) | 33 (65%) |  |  |  |  |  | 7.66 (5.4) |  |  |  |  |
| Muscatello M. et al. (2011)^49^ | 31.3 (4.6) | 23 (58%) |  |  |  |  |  |  |  | 34.6 (9.3) |  |  |
| Lin C. et al. (2010)^50^ | 38 | (58%) |  |  |  | 68.1 (14.3) | 25 |  |  |  |  |  |
| Li Y. et al.^51^ |  | 53 (45%) |  |  |  |  |  | 8.3 (8.8) |  |  | 87 (13.6) | 5.5 (0.9) |
| Jindal K. et al.^52^ |  | 34 (57%) |  |  |  |  |  |  |  | 46.7 (8.1) | 103.8 (13.0) |  |
| Kwon J. et al.1^53^ | 34.7 (11.4) | 212 (54%) |  |  |  | 63.2 (12.2) |  |  |  |  | 85.9 (13.1) | 4.9 (1.0) |
| SEROLA^54^ | 44.5 (8.0) | (65%) |  |  |  |  | 35.2 (9.9) |  |  |  | 61.6 |  |
| Lee B. et al.^55^ | 50.8 (2.6) | 26 (74%) |  |  |  |  |  | 20.0 (1.8) |  |  | 59.8 (3.4) |  |
| GiSAS^56^ |  |  |  |  |  |  |  |  |  |  |  |  |
| Muscatello M. et al. (2014)^57^ |  | 13 (33%) |  |  |  |  |  |  |  |  |  |  |

**References**

[1] Yamashita H, Mori K, Nagao M, Okamoto Y, Morinobu S, Yamawaki S. Effects of changing from typical to atypical antipsychotic drugs on subjective sleep quality in patients with schizophrenia in a Japanese population. J Clin Psychiatry 2004 Nov;65(11):1525-1530.

[2] Yamashita H, Mori K, Nagao M, Okamoto Y, Morinobu S, Yamawaki S. Influence of aging on the improvement of subjective sleep quality by atypical antipsychotic drugs in patients with schizophrenia: comparison of middle-aged and older adults. Am J Geriatr Psychiatry 2005 May;13(5):377-384.

[3] Zhong KX, Sweitzer DE, Hamer RM, Lieberman JA. Comparison of quetiapine and risperidone in the treatment of schizophrenia: A randomized, double-blind, flexible-dose, 8-week study. J Clin Psychiatry 2006 Jul;67(7):1093-1103.

[4] Lin CC, Chiu HJ, Chen JY, Liou YJ, Wang YC, Chen TT, et al. Switching from clozapine to zotepine in patients with schizophrenia: a 12-week prospective, randomized, rater blind, and parallel study. J Clin Psychopharmacol 2013 Apr;33(2):211-214.

[5] Wagner M, Quednow BB, Westheide J, Schlaepfer TE, Maier W, Kuhn KU. Cognitive improvement in schizophrenic patients does not require a serotonergic mechanism: randomized controlled trial of olanzapine vs amisulpride. Neuropsychopharmacology 2005 Feb;30(2):381-390.

[6] Chen JJ, Chan HY, Chen CH, Gau SS, Hwu HG. Risperidone and olanzapine versus another first generation antipsychotic in patients with schizophrenia inadequately responsive to first generation antipsychotics. Pharmacopsychiatry 2012 Mar;45(2):64-71.

[7] Riedel M, Muller N, Spellmann I, Engel RR, Musil R, Valdevit R, et al. Efficacy of olanzapine versus quetiapine on cognitive dysfunctions in patients with an acute episode of schizophrenia. Eur Arch Psychiatry Clin Neurosci 2007 Oct;257(7):402-412.

[8] Riedel M, Spellmann I, Strassnig M, Douhet A, Dehning S, Opgen-Rhein M, et al. Effects of risperidone and quetiapine on cognition in patients with schizophrenia and predominantly negative symptoms. Eur Arch Psychiatry Clin Neurosci 2007 Sep;257(6):360-370.

[9] Jakovljevic M, Pivac N, Mihaljevic-Peles A, Mustapic M, Relja M, Ljubicic D, et al. The effects of olanzapine and fluphenazine on plasma cortisol, prolactin and muscle rigidity in schizophrenic patients: a double blind study. Prog Neuropsychopharmacol Biol Psychiatry 2007 Mar 30;31(2):399-402.

[10] Strom BL, Faich GA, Reynolds RF, Eng SM, D'Agostino RB, Ruskin JN, et al. The Ziprasidone Observational Study of Cardiac Outcomes (ZODIAC): design and baseline subject characteristics. J Clin Psychiatry 2008 Jan;69(1):114-121.

[11] Strom BL, Eng SM, Faich G, Reynolds RF, D'Agostino RB, Ruskin J, et al. Comparative mortality associated with ziprasidone and olanzapine in real-world use among 18,154 patients with schizophrenia: The Ziprasidone Observational Study of Cardiac Outcomes (ZODIAC). Am J Psychiatry 2011 Feb;168(2):193-201.

[12] Freudenreich O, Henderson DC, Walsh JP, Culhane MA, Goff DC. Risperidone augmentation for schizophrenia partially responsive to clozapine: a double-blind, placebo-controlled trial. Schizophr Res 2007 May;92(1-3):90-94.

[13] Kryzhanovskaya L, Schulz SC, McDougle C, Frazier J, Dittmann R, Robertson-Plouch C, et al. Olanzapine versus placebo in adolescents with schizophrenia: a 6-week, randomized, double-blind, placebo-controlled trial. J Am Acad Child Adolesc Psychiatry 2009 Jan;48(1):60-70.

[14] Kemp DE, Correll CU, Tohen M, Delbello MP, Ganocy SJ, Findling RL, et al. Associations among obesity, acute weight gain, and response to treatment with olanzapine in adolescent schizophrenia. J Child Adolesc Psychopharmacol 2013 Oct;23(8):522-530.

[15] Berwaerts J, Cleton A, Rossenu S, Talluri K, Remmerie B, Janssens L, et al. A comparison of serum prolactin concentrations after administration of paliperidone extended-release and risperidone tablets in patients with schizophrenia. J Psychopharmacol 2010 Jul;24(7):1011-1018.

[16] Apiquian R, Fresan A, Ulloa RE, de la Fuente-Sandoval C, Herrera-Estrella M, Vazquez A, et al. Amoxapine as an atypical antipsychotic: a comparative study vs risperidone. Neuropsychopharmacology 2005 Dec;30(12):2236-2244.

[17] Harvey PD, Patterson TL, Potter LS, Zhong K, Brecher M. Improvement in social competence with short-term atypical antipsychotic treatment: a randomized, double-blind comparison of quetiapine versus risperidone for social competence, social cognition, and neuropsychological functioning. Am J Psychiatry 2006 Nov;163(11):1918-1925.

[18] Sacchetti E, Galluzzo A, Valsecchi P, Romeo F, Gorini B, Warrington L, et al. Ziprasidone vs clozapine in schizophrenia patients refractory to multiple antipsychotic treatments: the MOZART study. Schizophr Res 2009 Aug;113(1):112-121.

[19] Suzuki H, Gen K, Inoue Y. An unblinded comparison of the clinical and cognitive effects of switching from first-generation antipsychotics to aripiprazole, perospirone or olanzapine in patients with chronic schizophrenia. Prog Neuropsychopharmacol Biol Psychiatry 2011 Jan 15;35(1):161-168.

[20] Suzuki T, Uchida H, Watanabe K, Nomura K, Takeuchi H, Tomita M, et al. How effective is it to sequentially switch among Olanzapine, Quetiapine and Risperidone?--A randomized, open-label study of algorithm-based antipsychotic treatment to patients with symptomatic schizophrenia in the real-world clinical setting. Psychopharmacology (Berl) 2007 Dec;195(2):285-295.

[21] Kane JM, Osuntokun O, Kryzhanovskaya LA, Xu W, Stauffer VL, Watson SB, et al. A 28-week, randomized, double-blind study of olanzapine versus aripiprazole in the treatment of schizophrenia. J Clin Psychiatry 2009 Apr;70(4):572-581.

[22] Marder SR, Kramer M, Ford L, Eerdekens E, Lim P, Eerdekens M, et al. Efficacy and safety of paliperidone extended-release tablets: results of a 6-week, randomized, placebo-controlled study. Biol Psychiatry 2007 Dec 15;62(12):1363-1370.

[23] Findling RL, McKenna K, Earley WR, Stankowski J, Pathak S. Efficacy and safety of quetiapine in adolescents with schizophrenia investigated in a 6-week, double-blind, placebo-controlled trial. J Child Adolesc Psychopharmacol 2012 Oct;22(5):327-342.

[24] Gurpegui M, Alvarez E, Bousono M, Ciudad A, Carlos Gomez J, Olivares JM. Effect of olanzapine or risperidone treatment on some cognitive functions in a one-year follow-up of schizophrenia outpatients with prominent negative symptoms. Eur Neuropsychopharmacol 2007 Nov;17(11):725-734.

[25] Alvarez E, Ciudad A, Olivares JM, Bousono M, Gomez JC. A randomized, 1-year follow-up study of olanzapine and risperidone in the treatment of negative symptoms in outpatients with schizophrenia. J Clin Psychopharmacol 2006 Jun;26(3):238-249.

[26] Ciudad A, Olivares JM, Bousono M, Gomez JC, Alvarez E. Improvement in social functioning in outpatients with schizophrenia with prominent negative symptoms treated with olanzapine or risperidone in a 1 year randomized, open-label trial. Prog Neuropsychopharmacol Biol Psychiatry 2006 Dec 30;30(8):1515-1522.

[27] Zhang H, Li H, Shu L, Gu N, Wang G, Weng Y, et al. Double-blind comparison of ziprasidone and risperidone in the treatment of Chinese patients with acute exacerbation of schizophrenia. Neuropsychiatr dis treat 2011;7:77-85.

[28] Haas M, Unis AS, Armenteros J, Copenhaver MD, Quiroz JA, Kushner SF. A 6-week, randomized, double-blind, placebo-controlled study of the efficacy and safety of risperidone in adolescents with schizophrenia. J Child Adolesc Psychopharmacol 2009 Dec;19(6):611-621.

[29] Kahn RS, Schulz SC, Palazov VD, Reyes EB, Brecher M, Svensson O, et al. Efficacy and tolerability of once-daily extended release quetiapine fumarate in acute schizophrenia: a randomized, double-blind, placebo-controlled study. J Clin Psychiatry 2007 Jun;68(6):832-842.

[30] Hanssens L, L'Italien G, Loze JY, Marcus RN, Pans M, Kerselaers W. The effect of antipsychotic medication on sexual function and serum prolactin levels in community-treated schizophrenic patients: results from the Schizophrenia Trial of Aripiprazole (STAR) study (NCT00237913). BMC Psychiatry 2008;8:95.

[31] Taylor D, Hanssens L, Loze JY, Pans M, L'Italien G, Marcus RN. Preference of medicine and patient-reported quality of life in community-treated schizophrenic patients receiving aripiprazole vs standard of care: results from the STAR study. Eur Psychiatry 2008 Aug;23(5):336-343.

[32] Kerwin R, Millet B, Herman E, Banki CM, Lublin H, Pans M, et al. A multicentre, randomized, naturalistic, open-label study between aripiprazole and standard of care in the management of community-treated schizophrenic patients Schizophrenia Trial of Aripiprazole: (STAR) study. Eur Psychiatry 2007 Oct;22(7):433-443.

[33] Harvey PD, Sacchetti E, Galluzzo A, Romeo F, Gorini B, Bilder RM, et al. A randomized double-blind comparison of ziprasidone vs. clozapine for cognition in patients with schizophrenia selected for resistance or intolerance to previous treatment. Schizophr Res 2008 Oct;105(1-3):138-143.

[34] Okugawa G, Kato M, Wakeno M, Koh J, Morikawa M, Matsumoto N, et al. Randomized clinical comparison of perospirone and risperidone in patients with schizophrenia: Kansai Psychiatric Multicenter Study. Psychiatry Clin Neurosci 2009 Jun;63(3):322-328.

[35] Findling RL, Robb A, Nyilas M, Forbes RA, Jin N, Ivanova S, et al. A multiple-center, randomized, double-blind, placebo-controlled study of oral aripiprazole for treatment of adolescents with schizophrenia. Am J Psychiatry 2008 Nov;165(11):1432-1441.

[36] Voruganti LP, Awad AG, Parker G, Forrest C, Usmani Y, Fernando ML, et al. Cognition, functioning and quality of life in schizophrenia treatment: results of a one-year randomized controlled trial of olanzapine and quetiapine. Schizophr Res 2007 Nov;96(1-3):146-155.

[37] Cutler AJ, Kalali AH, Weiden PJ, Hamilton J, Wolfgang CD. Four-week, double-blind, placebo- and ziprasidone-controlled trial of iloperidone in patients with acute exacerbations of schizophrenia. J Clin Psychopharmacol 2008 Apr;28(2 Suppl 1):S20-8.

[38] Sacchetti E, Valsecchi P, Parrinello G, QUERISOLA G. A randomized, flexible-dose, quasi-naturalistic comparison of quetiapine, risperidone, and olanzapine in the short-term treatment of schizophrenia: the QUERISOLA trial. Schizophr Res 2008 Jan;98(1-3):55-65.

[39] Maat A, Cahn W, Gijsman HJ, Hovens JE, Kahn RS, Aleman A. Open, randomized trial of the effects of aripiprazole versus risperidone on social cognition in schizophrenia. Eur Neuropsychopharmacol 2014 Apr;24(4):575-584.

[40] Chang JS, Ahn YM, Park HJ, Lee KY, Kim SH, Kang UG, et al. Aripiprazole augmentation in clozapine-treated patients with refractory schizophrenia: an 8-week, randomized, double-blind, placebo-controlled trial. J Clin Psychiatry 2008 May;69(5):720-731.

[41] Fleischhacker WW, Heikkinen ME, Olie JP, Landsberg W, Dewaele P, McQuade RD, et al. Effects of adjunctive treatment with aripiprazole on body weight and clinical efficacy in schizophrenia patients treated with clozapine: a randomized, double-blind, placebo-controlled trial. Int J Neuropsychopharmcol 2010 Sep;13(8):1115-1125.

[42] Nose M, Accordini S, Artioli P, Barale F, Barbui C, Beneduce R, et al. Rationale and design of an independent randomised controlled trial evaluating the effectiveness of aripiprazole or haloperidol in combination with clozapine for treatment-resistant schizophrenia. Trials 2009;10:31.

[43] Barbui C. Accordini S. Nose M. Stroup S. Purgato M. Girlanda F. Esposito E. Veronese A. Tansella M. Cipriani A. CHAT (Clozapine Haloperidol Aripiprazole Trial) Study Group. Aripiprazole versus haloperidol in combination with clozapine for treatment-resistant schizophrenia in routine clinical care: a randomized, controlled trial. J Clin Psychopharmacol 2011 Jun;31(3):266-273.

[44] Cipriani A, Accordini S, Nose M, Purgato M, Girlanda F, Tansella M, et al. Aripiprazole versus haloperidol in combination with clozapine for treatment-resistant schizophrenia: a 12-month, randomized, naturalistic trial. J Clin Psychopharmacol 2013 Aug;33(4):533-537.

[45] De Hert M, Mittoux A, He Y, Peuskens J. Metabolic parameters in the short- and long-term treatment of schizophrenia with sertindole or risperidone. Eur Arch Psychiatry Clin Neurosci 2011 Jun;261(4):231-239.

[46] Tybura P, Samochowiec A, Beszlej A, Grzywacz A, Mak M, Frydecka D, et al. Some dopaminergic genes polymorphisms are not associated with response to antipsychotic drugs in schizophrenic patients. Pharmacol Rep 2012;64(3):528-535.

[47] Schreiner A, Niehaus D, Shuriquie NA, Aadamsoo K, Korcsog P, Salinas R, et al. Metabolic effects of paliperidone extended release versus oral olanzapine in patients with schizophrenia: a prospective, randomized, controlled trial. J Clin Psychopharmacol 2012 Aug;32(4):449-457.

[48] Ghaleiha A, Honarbakhsh N, Boroumand MA, Jafarinia M, Tabrizi M, Rezaei F, et al. Correlation of adenosinergic activity with superior efficacy of clozapine for treatment of chronic schizophrenia: a double blind randomised trial. Hum Psychopharmacol 2011 Mar;26(2):120-124.

[49] Muscatello MR, Bruno A, Pandolfo G, Mico U, Scimeca G, Di Nardo F, et al. Effect of aripiprazole augmentation of clozapine in schizophrenia: a double-blind, placebo-controlled study. Schizophr Res 2011 Apr;127(1-3):93-99.

[50] Lin CH, Kuo CC, Chou LS, Chen YH, Chen CC, Huang KH, et al. A randomized, double-blind comparison of risperidone versus low-dose risperidone plus low-dose haloperidol in treating schizophrenia. J Clin Psychopharmacol 2010 Oct;30(5):518-525.

[51] Li Y, Li H, Liu Y, Yan X, Yue Y, Qian M. Comparison of quetiapine and risperidone in Chinese Han patients with schizophrenia: results of a single-blind, randomized study. Curr Med Res Opin 2012 Oct;28(10):1725-1732.

[52] Jindal KC, Singh GP, Munjal V. Aripiprazole versus olanzapine in the treatment of schizophrenia: a clinical study from India. Int J Psychiatry Clin Pract 2013 Feb;17(1):21-29.

[53] Kwon JS, Mittoux A, Hwang JY, Ong A, Cai ZJ, Su TP. The efficacy and safety of 12 weeks of treatment with sertindole or olanzapine in patients with chronic schizophrenia who did not respond successfully to their previous treatments: a randomized, double-blind, parallel-group, flexible-dose study. Int Clin Psychopharmacol 2012 Nov;27(6):326-335.

[54] Ernst Nielsen R, Odur F, Ostergaard T, Munk-Jorgensen P, Nielsen J. Comparison of the effects of Sertindole and Olanzapine on Cognition (SEROLA): a double-blind randomized 12-week study of patients diagnosed with schizophrenia. Ther adv psychopharmacol 2014 Feb;4(1):4-14.

[55] Lee BJ, Lee SJ, Kim MK, Lee JG, Park SW, Kim GM, et al. Effect of aripiprazole on cognitive function and hyperprolactinemia in patients with schizophrenia treated with risperidone. Clin Psychopharmacol Neurosci 2013 Aug;11(2):60-66.

[56] Parabiaghi A, D'Avanzo B, Tettamanti M, Barbato A, GiSAS Study G. The GiSAS study: rationale and design of a pragmatic randomized controlled trial on aripiprazole, olanzapine and haloperidol in the long-term treatment of schizophrenia. Contemp Clin Trials 2011 Sep;32(5):675-684.

[57] Muscatello MR, Pandolfo G, Mico U, Lamberti Castronuovo E, Abenavoli E, Scimeca G, et al. Augmentation of clozapine with ziprasidone in refractory schizophrenia: a double-blind, placebo-controlled study. J Clin Psychopharmacol 2014 Feb;34(1):129-133.
